# Signs and symptoms in children with a serious infection: a qualitative study

**DOI:** 10.1186/1471-2296-6-36

**Published:** 2005-08-26

**Authors:** Ann Van den Bruel, Rudi Bruyninckx, Etienne Vermeire, Peter Aerssens, Bert Aertgeerts, Frank Buntinx

**Affiliations:** 1 Department of General Practice, Katholieke Universiteit Leuven, Kapucijnenvoer 33 Blok J, 3000 Leuven, Belgium; 2 Department of General Practice, Universtaire Instelling Antwerpen, Universiteitsplein 1, 2610 Wilrijk, Belgium; 3 Department of Pediatrics, Virga Jesseziekenhuis, Stadsomvaart 11, 3500 Hasselt, Belgium; 4 Department of General Practice, Universiteit Maastricht, Postbus 616, 6200 MD Maastricht, The Netherlands

## Abstract

**Background:**

Early diagnosis of serious infections in children is difficult in general practice, as incidence is low, patients present themselves at an early stage of the disease and diagnostic tools are limited to signs and symptoms from observation, clinical history and physical examination. Little is known which signs and symptoms are important in general practice. With this qualitative study, we aimed to identify possible new important diagnostic variables.

**Methods:**

Semi-structured interviews with parents and physicians of children with a serious infection. We investigated all signs and symptoms that were related to or preceded the diagnosis. The analysis was done according to the grounded theory approach. Participants were recruited in general practice and at the hospital.

**Results:**

18 children who were hospitalised because of a serious infection were included. On average, parents and paediatricians were interviewed 3 days after admittance of the child to hospital, general practitioners between 5 and 8 days after the initial contact.

The most prominent diagnostic signs in seriously ill children were changed behaviour, crying characteristics and the parents' opinion. Children either behaved drowsy or irritable and cried differently, either moaning or an inconsolable, loud crying. The parents found this illness different from previous illnesses, because of the seriousness or duration of the symptoms, or the occurrence of a critical incident. Classical signs, like high fever, petechiae or abnormalities at auscultation were helpful for the diagnosis when they were present, but not helpful when they were absent.

**Conclusion:**

behavioural signs and symptoms were very prominent in children with a serious infection. They will be further assessed for diagnostic accuracy in a subsequent, quantitative diagnostic study.

## Background

In general practice, the incidence of acute infections is high. The yearly incidence can be as high as 41%, with acute upper respiratory infections as the most frequent diagnosis and highest incidence rates in children less than 1 year old[1].

In contrast, serious and possibly life-threatening infections are rare, their yearly incidence in children being estimated at 1.5%[2]. The most frequent diagnoses are pneumonia, sepsis, meningitis, pyelonephritis and bacterial gastro-enteritis [3-6]. Other infections, such as osteomyelitis, cellulitis and septic arthritis are even less frequent.

This low incidence and the similarities in the initial presentation make it difficult to distinguish these children from their peers with a non-serious, mostly self-limiting infection.

Still, early diagnosis of a serious infection is important to avoid delay in treatment and improve prognosis [7-11].

Signs and symptoms are the first and most readily available diagnostic tools for the general practitioner. They are the basis of subsequent decisions, such as referral, additional testing or prompt treatment.

However, little is understood about these signs and symptoms in general practice. Prior diagnostic accuracy studies on serious infections in children were predominantly conducted in hospital populations, in very young children or included laboratory or radiology tests results [12-15]. For example, the Yale Observation Scale uses observational information, but was constructed and validated in a hospital setting[16]. The same applies for the Young Infant Observation Scale, which also uses observational information, but which is designed for hospitalised children aged less than 8 weeks old[17]. None of the above was evaluated in general practice, to our knowledge only a few studies were performed in general practice on this subject [18-20]. These studies do not provide quantitative measures of accuracy and cover only meningitis, instead of the entire group of serious infections. Other studies about signs and symptoms of children with a serious infection in primary care were carried out in developing countries in a population that is not comparable to that in Western Europe [21-24].

As the value of a diagnostic test depends on the setting in which it is being used[25,26], the results of these studies can not simply be transferred to general practice and therefore new research is needed.

A number of signs and symptoms have been described in the past as being related to the diagnosis of a serious infection in children. All available information, however, indicates that they are insufficient to reliably diagnose or exclude a serious infection[27,28]. We therefore conducted this qualitative study to generate hypotheses, as part of a large diagnostic study about signs and symptoms of serious infections in children, seen in general practice. With this qualitative study, we intended to identify new signs and symptoms, additional to those found in textbooks or main articles[29] and promising for use in general practice. It is our intention to quantitatively estimate their diagnostic characteristics in a subsequent prospective study.

## Methods

We selected a theoretical sample[30] from all children that were admitted with a serious infection to one large regional hospital in the east of Flanders, Belgium. The sample was intended to consist of four children with one of the following infections: pneumonia, sepsis, meningitis, pyelonephritis and complicated gastro-enteritis. Of the latter, two children with a complicated viral gastro-enteritis and two with a bacterial gastro-enteritis were included. Within each subgroup, the children were included consecutively.

This purposive sampling was performed to ensure a wide range of possible signs and symptoms, different perspectives from parents and physicians from both general practice and specialist care[31]. This sampling procedure is characteristic for qualitative studies and should not be mistaken with the classical sampling procedures for a diagnostic accuracy study.

### Design

The study was performed between March and October 2003.

The children's parents were invited for a semi-structured interview. We also interviewed the paediatrician who had admitted the child and the general practitioner if he or she had seen the child before admittance. The interviews were carried out as soon as possible after admittance to the hospital or after consultation at the general practice, in order to minimise recall bias.

Our main point of interest was the identification of signs and symptoms that were present in all cases or in all cases of one of the diagnostic categories.

Informed consent was signed by all parents. The study was approved by the Ethical Review Board of the Katholieke Universiteit Leuven.

The interviews were semi-structured[32]: a framework for the interview was set out, covering different areas of possible signs and symptoms. Besides this framework, open ended questions about the history of the disease or any other information the interviewee wanted to communicate, were included in order to retrieve possible new items. Before the start of the pilot study, both the content of the interview and the phrasing of the questions were reviewed by experts in qualitative research and in paediatrics. Changes were made according to their remarks. The interview was piloted twice by the principal investigator with two GPs who had recently seen a child with a serious infection. After these interviews, the GPs were asked to evaluate the content of the questions, the method of performing the interview and the emotions that it possibly evoked. No more substantial changes were needed at this stage.

The interviews were performed by three experienced interviewers and the principal investigator. The interviewers were instructed about the goal of the study and trained during a practice session. All interviews were recorded on tape and transcribed before starting the analysis. During the interview, the atmosphere, non-verbal communication and the possible effect of other people that assisted were described by the interviewers. The framework of the interview is illustrated in table 1.

**Table 1:** framework of the interviews

1. Opening of the interview: the purpose of the study was repeated and informed consent specifically asked again.
2. Start of the interview with questions on the child's name and the relation of the interviewee to the child.
3. Open questions on the illness episode. Interviewees were asked to tell in their own words what had happened.
4. More directed questions on the start of the illness, the evolution of the illness and possible symptoms.
5. Open questions on the decisions and actions of the interviewee taken during the course of the illness, including the reasons for these decisions.
6. Final open question: whether the interviewee had anything to add to the interview.

Analysis was performed according to the pragmatic variant of the grounded theory approach[33], by which new themes are identified from the data alongside those that were already anticipated from the outset[34]. Every interview was independently analysed by two investigators. The principal investigator analysed all interviews and two other investigators each analysed half of the interviews. Individual codes were assigned to the text by each investigator. The process was iterative, as sampling and data collection were guided by the emerging analysis. During a consensus session, all codes were compared and disagreements were reviewed with the data at hand. This resulted in a set of themes that were striking and seemed important diagnostic features, either across different diagnostic subgroups or in one diagnostic subgroup only. These themes were than translated in hypotheses for subsequent quantitative tests.

## Results

In total, 22 children with a serious infection were eligible for the study. Three children were excluded: 1 because a second reading of the X-ray refuted the diagnosis of pneumonia and 2 because the delay between admittance and the first contact with the researcher exceeded 5 days. All parents gave their consent, as well as all paediatricians. In 9 cases, the children were seen by their general practitioner before admittance to the hospital. All GPs agreed to participate. One child was excluded after the interview, because the audiotape was unintelligible. Interviews with the parents and paediatricians of 18 children and with the GPs of 9 children with a serious infection were thus available for analysis. We could include only 2 cases of meningitis within the study period instead of the expected 4 cases. But as no new items were identified in the last two interviews, the data collection was ended.

### Demographics

The mean age of the children was 2.5 years (ranging from 14 days to 11 years) and 9 of the 18 children were girls.

As mentioned above, we included 4 children from each diagnostic category, except for meningitis, from which only 2 cases were included.

Average time between admittance to the hospital and the interview with the parents was 2.8 days (range from 1 to 8 days) and to the interviews with the paediatricians was 3.4 days (range from 1 to 8 days). General practitioners were interviewed 7.8 days (range from 1 to 14 days) after seeing the child. The interviewers took notes on the atmosphere of the interview, and of the people present. In most cases the atmosphere was relaxed, especially with the parents; physicians sometimes seemed hurried. The mother of the child was present in the majority of the interviews with the parents. Any non-verbal signs important for the context of the interviews were noted as well. These indicated that most interviewees were motivated and at ease, although one paediatric resident was sceptical on the purpose of the study on three occasions.

#### Behaviour

Behavioural changes were almost systematically mentioned. They were twofold: on one hand some children were very weak or drowsy; on the other hand there were children who were irritable. Changes in behaviour were mentioned by both parents and physicians, but very meticulously illustrated by the parents as they compared it to the normal behaviour of the child.

### Drowsiness, as described by parents

'*It seemed as if there was no life in him anymore*': this quote was often repeated by different parents, in almost exact the same words. Their children were unusually quiet, did not play nor talk. Some children could not get up, and had to be fed lying in bed. Several children were lying in bed or on the sofa, with their eyes closed without actually sleeping. Many children did not laugh anymore, even when they were being played with.

They were all not in their usual self, on several occasions phrased as '*This was no longer my child*.'

[Parent, child with meningococcal sepsis, 1 year old] *"It was really different from before. In the morning she was lying in her playpen and she stayed so still, she really wasn't her normal self."*

[Parent, child with pneumonia, 5 years old] *"He was lying there with his eyes closed, but when I asked him something, he answered me, so he wasn't sleeping, but he felt so miserable, I guess. It was frightening, because normally he is such a lively child, and now he couldn't even watch television."*

[Parent, child with sepsis, 8 months old] *"He was very passive and normally he is a very cheerful baby, he is always laughing and always content. Now he was not laughing anymore and that's why I knew there was something wrong."*

### Drowsiness, as described by physicians

Physicians also found it abnormal when a child did not react to strangers, and especially when it was manipulated for a clinical examination or a blood sample. Sometimes, general physicians noticed that the child was not behaving like it did on previous occasions, as they have, more than a paediatrician, a long-term relationship with the family.

[General practitioner, child with pneumonia, 2 years old] *"She was so drowsy. Normally she is a very lively child."*

[General practitioner, child with meningococcal sepsis, 1 year old] *"I went there, and when I arrived I saw immediately that it was serious. The child was so passive!"*

[General practitioner, child with pneumonia, 2 years old] *"For a two year old, she let herself be examined too easily. Normally we expect some more resistance."*

[Paediatrician, child with viral gastro-enteritis with dehydration, 2 years old] *"Almost no reactions. While taking the blood sample he did not react either."*

### Irritability

Some children behaved more irritable, for example they started crying and could not be consoled, slept less than usual or woke up during the night. Other children were irritable when they were examined by a physician.

[Parent, child of 14 days old with pyelonephritis] *"He almost did not sleep, very shortly in the afternoon, one hour or so. Normally he sleeps more than four hours; but now he woke after one hour and he did not sleep after since."*

[Parent, child with sepsis, 1 year old] *"He wanted to go to bed more often, but he could not sleep. And normally, when we put him between us he becomes very quiet, but that did not help this time. Taking him up did not help either, and when he is crying with his dummy in his mouth, then it is not right."*

[Paediatrician, child with pyelonephritis, 14 days old] *"He was irritable when he was touched."*

### Crying characteristics

The cry was often found to be different than before, as expressed by their parents, or to be striking, as expressed by the physicians. The way this crying was different, was parallel to the other behavioural changes with children who were drowsy and other who were irritable.

Some children were crying in a nagging, quiet way, with less force than usual. Other children were crying louder than usual, could not be consoled or had a pinching cry.

[General practitioner, child with pneumonia, 2 years old] *"Not really crying, but moaning. And she had been doing that at home as well, according to the grandmother, for the last two days."*

[Parent, child with meningitis, 2 months old] "*Wednesday at midnight, he suddenly started shrieking. Normally he cries a little and then you take him, but now he was really shrieking. He wouldn't be quiet with a bottle or anything."*

[Paediatrician, child with sepsis, 8 months old] "*The pinching cry was the only thing that struck me. He didn't seem very sick."*

#### Abnormal illness

Apart from behavioural changes, parents found the illness itself different from previous illnesses. Symptoms could be different than usual or the duration of the illness was longer.

[Child, with bacterial gastro-enteritis, 11 years old] *"The diarrhoea, I have never had it so seriously. I really panicked."*

[Parent, with bacterial gastro-enteritis, 4 years old] *"His weight was 17 kg and when I weighed him that morning it was only 15.3 kg. I found he had lost too much weight in 2 days."*

[Parent, child with pneumonia, 5 years old] *"It went on and on, I thought this can not be right."*

[Parent, child with pyelonephritis, 2 years old] *"She did not eat anything and she could not hold down her drinks; then you know it is not right."*

[Parent, child with pneumonia, 5 years old] *"Three days of fever is perfectly normal, even five days; but after these five days I thought it was abnormal."*

On several occasions, there was an incident that had never happened before and made the parents anxious or made them decide to seek help immediately. These incidents were variable, a few children suddenly became very pale and limp, and another vomited in her sleep without waking up. One child with viral meningitis became unconscious at school.

[Parent, child with sepsis, 2 years old] "*The moment he was sitting on my lap and suddenly collapsed, I was really frightened and came here immediately. At that moment I just knew it was more than just a cold."*

[Parent, child with meningitis, 2 months old] *"I did not trust it because he wasn't himself. But then he suddenly became very pale after I had cooled him, and it was really strange."*

#### Parents' opinion

Several physicians said that they were guided by the opinion of the parents in their judgement. In addition to other signs, the parents' anxiety or statement that it was an abnormal situation made them cautious. In fact, when we asked them which was the most striking sign or symptom, several physicians answered that it was the parents' opinion.

[General practitioner, child with meningococcal sepsis, 1 year old] *"The mother rang back very quickly after a few hours, which is very odd for her. She is very capable of handling a situation like that; normally she doesn't mind a child having a fever for two or three days."*

[Paediatrician, child with meningitis, 2 months old] *"Especially the mother saying that she did not know the child like that."*

#### Classical signs

Almost all children with a serious infection had a high fever of over 39°C (102 degrees Fahrenheit), except one infant of two weeks old with a pyelonephritis and one toddler with a viral meningitis. The latter two had a body temperature of over 38°C (100.4 degrees Fahrenheit).

Other classical signs as described in textbooks were present in some cases but not in all. For example, neck stiffness, petechiae, crepitations or signs of dehydration, were present in some children and then led towards the diagnosis, but not all children presented with any of these suspected signs. The absence of a sign did not necessarily mean the absence of disease and some classical signs would not appear in all children with a certain disease, as was shown by the management decision of the treating physicians in our sample.

This was especially the case for signs from auscultation and the diagnosis of pneumonia, which were not present in any child with pneumonia. Only one child with pneumonia had a cough at the moment of admittance. Two children with pneumonia started coughing later during the hospital stay.

Other signs such as vomiting or the absence of signs of upper respiratory tract infection could be observed in several of the children with a serious infection. However, in some of the very sick children no classical signs could be found, which was disturbing the physicians on itself.

[General practitioner, child with pneumonia, 2 years old] *"Nothing specific, I did not find anything and that child was really sick. I could not find anything, except a sensitive tummy, and then you think: this can not be right."*

#### Disease specific

All children with pneumonia had some sign concerning the breathing. Some children had a higher breathing rhythm, some had superficial breathing, only one child with pneumonia had a dry cough.

Of the children with pyelonephritis, two started wetting their pants again, after they had been toilet-trained long before. The other two children with pyelonephritis in the sample were still in nappies, so it was difficult to observe urination signs.

Loss of weight was prominent in children with gastro-enteritis, but this is to be expected, as it is a criterion for admittance to the hospital.

The children with sepsis all had petechiae but one. In one child, the petechiae were rather large and expanding, in the other two, there were only a few spots that could easily have been missed. In these last two cases, none of the parents had noticed the spots; they were only seen after careful inspection at the emergency department.

For the diagnosis of meningitis, only two cases could be included, both of viral aetiology. The first child was a baby of two months old, who cried in a different way than before and was difficult to console. The second child was a toddler who fell asleep at school and could not be awakened unless with pain stimuli. None of these children had signs of meningeal irritation.

## Discussion

Serious infections in children are an important topic in primary care, because of the related mortality and morbidity [35-38]. This was reflected in the high response ratios to our invitation to participate in the study, as all but one parent and one doctor agreed to participate.

The diagnosis of these infections can be difficult, especially in primary care, where the disease is still in an early stage and incidence is low. This, together with the relative inaccessibility of more invasive diagnostic procedures, makes it a difficult challenge for the general practitioner and cases can be missed[39]. In a qualitative study about the diagnosis and management of children suspected with meningitis[40], general practitioners found it difficult to reach a diagnosis and stated they relied upon intuitive rather than systematic methods. In order to be transmissible to younger generations, however, intuition has to be translated in evidence.

In our study, time between admission or consultation and interview was very short (between 2.8 and 7.8 days), especially when this is compared to another qualitative study about the diagnosis of meningitis in primary care, where the mean interval between case and interview was 61 weeks[41]. Even so, in our study, paediatricians and GPs frequently consulted their file, which indicates that although physicians were willing to answer the questions accurately they had difficulties in remembering certain details.

The interviews were mostly taken in a relaxed atmosphere, although GPs and paediatricians often had limited time available. Parents were very motivated for the study in both patient groups. One paediatric resident was sceptical about the study at the beginning, but became more convinced at a later stage. The short time frame, the relaxed and open atmosphere and the motivation and interest for the study strengthen the validity of our study results. Our study is limited by the fact that, although the primary aim was to explore the clinical presentation of children with a serious infection in general practice, we were able to interview only 9 general practitioners. It may be reasonable to assume, however, that the information given by the parents is useful in general practice as well, as physicians should be very sensitive to the information given by the parents during history taking. The information given by parents can be influenced by their educational status, marital status, number of children etcetera. Unfortunately, we do not have any data to check this in our sample.

Our clinical findings are partly in concordance with findings from other studies, but some are different.

Parents can describe their child's behaviour very accurately and can compare this to the normal behaviour of the child and to previous illnesses. The level of detail in which these descriptions were made, was very striking.

Some children were drowsy and weak, more than they were during previous illnesses. Children cried in a different way than they normally did, moaning, nagging, a cry without force. Other children were irritable, cried louder than usual and could not be consoled. A pinching cry was noticed by physicians as well.

Observation variables have been described before [42-44], but these were mainly variables from the doctor's own observations. Our findings suggest that physicians should be very sensitive to what parents are telling them and add this information to their own observations. For this, general practitioners are in a favourable position, as they have a long-term relationship with their patients and can relate this new information to previous contacts.

Parents found this illness different from previous illnesses. Symptoms were more serious, the duration was longer and sometimes there was a critical incident which caused anxiety or warranted further actions. Some physicians were very sensitive to the opinion of the parents, especially if they knew the family before, and saw a difference in reaction of the parents compared to previous occasions.

Serious infections tend to present with high fever. This has been shown before, for example by Hewson[45], Bleeker[46] and Kuppermann [47-49]. Other studies have found no relation between high temperature and serious infections[50,51]. However, it is possible that fever is a valuable sign in an unselected population such as in general practice, but of less value in a selected population, such as children seen at an emergency department. This certainly needs to be addressed in our subsequent, quantitative diagnostic study.

Secondly, signs can be asymmetric, i.e. their presence has more diagnostic power than their absence or vice versa. This has also been demonstrated before [52-55]. In our study, it seems that some 'classical' signs are very informative when they are present, while the absence of these signs provided almost no information to rule out the suspected disease.

Besides these more generic characteristics, we also found disease-specific characteristics that could be important in the diagnosis of these infections. Signs on breathing pattern, urinary symptoms, rashes were present in most of these cases and certainly should be explored further in a general practice setting. Most of these signs have been proven to be of value before [56-58], but hardly ever in general practice [19,59,17]. The diagnostic accuracy of these signs and symptoms could be addressed in a future study.

Overall, this study did not aim to give 'hard' evidence on tests for the diagnosis of serious infections in children. The reason for performing the study was the lack of evidence in general practice; possible new or different signs had to be explored. The results of this study indicate signs that may be apparent in general practice. This qualitative study provides hypotheses, which can be tested in a quantitative study.

## Conclusion

This study has revealed several interesting diagnostic signs about serious infections in children in general practice, especially changed behaviour, crying characteristics, parents' opinion and some classical signs.

These hypotheses can be tested in a prospective, quantitative study to determine their diagnostic accuracy, during which possible asymmetries can be evaluated.

## Competing interests

The author(s) declare that they have no competing interests.

## Authors' contributions

AVDB contributed to the design and protocol of the study, participated in the data collection, was the primary researcher of the analysis and drafted the manuscript.

RB contributed to the protocol and made a substantial contribution to the analysis. EV provided assistance with the analysis and the manuscript. BA helped with the design of the study and the manuscript. PA helped with the design, the protocol and the data collection. FB conceived the study, set up the design, guided and participated in the analysis and revised the manuscript.

All authors read and approved the manuscript.

## Pre-publication history

The pre-publication history for this paper can be accessed here:


